# A Meta-Analysis of Studies of Treatments for Feline Urine Spraying

**DOI:** 10.1371/journal.pone.0018448

**Published:** 2011-04-15

**Authors:** Daniel S. Mills, Sarah E. Redgate, Gary M. Landsberg

**Affiliations:** 1 Animal Behaviour, Cognition and Welfare Group, Department of Biological Sciences, University of Lincoln, Lincoln, United Kingdom; 2 North Toronto Animal Clinic, Thornhill, Ontario, Canada; Tulane University, United States of America

## Abstract

Feline urine spraying inside the home is a common problem behaviour that owners seek advice for from veterinarians. Individual trials relating to a variety of interventions produce variable results, and to date, no consensus on the value of different treatments has emerged. This study therefore aimed to meta-analyse, current data from appropriate published clinical trials that evaluate treatments for feline urine spraying.

Inclusion and exclusion criteria for study selection were predefined and methodological quality was assessed by two independent reviewers. Ten studies in nine publications that either evaluated pharmacotherapy or pheromonatherapy (the use of a synthetic analogue of the F3 facial fraction in the cat) were suitable for analysis. There was a significant (P<0.001) association between the use of any intervention and the number of cats that ceased or reduced urine spraying by at least 90%. Analysis by intervention type indicated that fluoxetine, clomipramine and pheromonatherapy may each assist in managing urine spraying beyond a placebo based intervention.

This is the first time meta-analytical techniques have been used and reported to evaluate the efficacy of interventions used in veterinary behavioural medicine, and it has established confidence in the value of both conventional treatments (pharmacotherapy) and a more recently developed treatment modality (pheromonatherapy) as an adjunct to the management of this problem. It is suggested that future research into treatment efficacy for this problem uses the benchmark standard of randomised, controlled trials lasting for at least 8 weeks, with the outcome criteria of cessation of feline urine spraying or reduction by at least 90%.

## Introduction

Urine spraying forms a normal part of the cat's behavioural repertoire and can broadly be categorized as either sexual (associated with reproductive function) or reactional (associated with threats to resources) marking [1]. It is shown by both sexes, all breeds and occurs irrespective of neutering, with approximately 10% of neutered males and 5% of spayed females exhibiting the behaviour [1], [2], [3].

The behavioural sequence observed may vary subtly between cats. In general, the cat will turn its back on the area of choice, raise the tail and arch the back, then spray a variable quantity of urine onto a vertical surface, whilst spraying the tail may also quiver [4]. Vertical surfaces in the house are commonly sprayed areas, often when they are near access points or windows. Owners also report that targets include objects on the floor such as boxes or bags and electrical items including plug sockets and household appliances.

The number of areas sprayed differs between individuals with some cats limiting spraying to one place, for instance a door frame while others spray in multiple sites around the home. The frequency of spraying episodes varies between households ranging to in excess of 63 sprays a week [2], [5], [6]. The behaviour frequently becomes a problem for the owner or carer and in extreme cases may be the sole reason for relinquishment [7], [8]. Data from veterinary referrals to registered “Pet Behaviour Counsellors” show that urine spraying is one of the most frequently recognised behaviour problems for which cat owners seek advice [9].

Feline lower urinary tract disorders have been associated with the development of spraying and many behavioural and environmental factors have also been implicated [6], [10]. The number of cats living in the household and the frequency of inter cat aggression have been identified as risk factors, as have environmental triggers such as a substantial changes in the household, changes in routine or presence of neighbouring cats [2], [11], [12].

Traditional management of the problem has included neutering and or treatment with progestins [13], although the latter are no longer generally recommended due to their side effects. Current strategies advocate cleaning regimes for the urine and behavioural modification to remove any specific triggers alongside specific psychopharmacological and non-pharmacological interventions such as use of the feline facial pheromone fraction in the environment [14]. Suggested psychopharmacological treatments include benzodiazepines, azapirones, tricyclic antidepressants and selective serotonin reuptake inhibitors [1], [15], [16], [17], [18], [19].

To date a small number of randomised control trials and one-group, uncontrolled trials have been carried out to evaluate the effectiveness of intervention on the control of urine spraying. The evidence indicates that none of the currently available interventions are successful in completely resolving the behaviour in all spraying cats, for this reason treatment outcome is often defined in terms of number of cats that cease spraying and / or reduce spraying beyond a certain rate. For example, Pryor *et al.,* [18]) defines success as cessation or a 90% reduction of signs whereas, Mills and Mills [6] report numbers that cease and numbers that reduce.

This report aims to synthesize the current data from published clinical trials that evaluate treatments for feline urine spraying. A meta-analytical technique is used to evaluate peer reviewed studies with appropriate data in order to discern the influence that non-behavioural intervention methods have on the incidence of either the cessation of urine spraying or its reduction.

## Materials and Methods

### Ethics Statement

This project was approved by the University of Lincoln local ethics committee. A review protocol was not preregistered.

### Search methods

Published reports of clinical trials evaluating an intervention for feline urine spraying were collected through a comprehensive search of three electronic databases ISI Web of Knowledge, Ingenta Connect and Science Direct and one web search engine Google Scholar. The search terms used were: Urine spraying, Urine marking, Cat, Feline, Behaviour/Behavior. For the databases the terms were entered into the topic section (ISI Web of Knowledge) or through an advanced search and terms were entered into the title, abstract, keywords section (Ingenta Connect and Science Direct), without a publication date restriction. Databases were accessed on the 25^th^ April and 1^st^ May 2009. In addition, the references of all identified studies were inspected for additional studies.

### Study selection

Inclusion and exclusion criteria for study selection were predefined, with some selected specifically to minimise the risk of bias. All studies met the following inclusion criteria (1) the study was published as a peer reviewed publication; (2) the study provided sufficient information to extract representative data. We excluded studies for the following reasons (1) cats were showing sexual and not reactional spraying; (2) case studies; (3) follow up studies (4) brief reporting of materials and methods (5) cats showing horizontal urination.

All papers analysed were classified according to their level of evidence and risk of bias as defined by the Centre for Evidence Based Medicine, Oxford, UK [20]. Two reviewers (SR and GL) independently rated the studies, rating results were compared, and where differences were noted, they were discussed and reconciled.

#### Definition of a successful treatment outcome

The definition of a successful treatment outcome varied between studies and included, the number of cats that ceased or reduced spraying by 90% [18], [21] or the number of cats that ceased [2], [6]. In addition, to the numbers that cease a proportion of studies also reported the number of cats that reduced spraying relative to baseline [2], [5], [6]. To compare all studies the number of cats that either ceased or reduced spraying by at least 90% was used as a primary outcome measure as it described the tightest criterion for success among studies. However, as not all the cats ceased or reduced spraying by at least 90% a secondary outcome measure was formulated of the number of cats that reduce spraying from baseline levels.

### Data Analysis

Data were extracted from all published studies; in addition, unpublished raw data were available from one study by the paper author (D Mills). When full subject data were presented in tabular format these data were used to extract the required information. Missing data were taken into account when reporting total sample sizes.

Meta-analysis is a statistical procedure for combining data from multiple studies in order to identify a common effect of the treatment.

Data were analyzed using Comprehensive Meta-analysis Software version 2 (Biostat, Englewood, NJ, USA).

The potential heterogeneity among studies was tested using a Cochran test. In case of evidence of significant heterogeneity a random effects model was used. Alternatively, a fixed effects model was used when heterogeneity was not evident [22].

The possible influence of publication bias was evaluated by means of a funnel plot where log-transformed odds ratios were plotted against standard errors.

#### Estimation of placebo effect

In order to allow us to include studies without placebo control and assess if these studies were biased compared to those with a placebo control, a placebo effect was estimated. The placebo effect was evaluated on randomized controlled trials with a negative control. The global placebo effect was calculated with events rate and its 95% confidence interval.

#### Analysis of the primary outcome

A notable caveat for this analysis is the lack of studies with a placebo control group. In an attempt to remedy this we used the information from two studies with a placebo group [6], [18] to generate an estimate of the effect size of the placebo. This effect size was then used in an analysis of all studies in order to compare interventions. Event rate for the treated group of each study was compared against the estimated event rate for the placebo group. These events rates were calculated in order to produce a 95% odds ratio and its 95% confidence interval for each study and for the global treatment effect.

The odds ratios compare the likelihood that the subject exposed to the treatment will develop the outcome compared to a subject that is not exposed.

Further separate analyses were then performed to determine the influence of intervention type (fluoxetine, clomipramine and synthetic feline facial pheromone F3).

#### Analysis of the secondary outcome

It was expected that the majority of studies which simply reported a reduction in spraying would have used the synthetic feline facial pheromone F3. Therefore an additional predefined analysis was performed to examine the influence of pheromones on a reduction in urine spraying in comparison to baseline levels. Event rate and its confidence interval were calculated for the treated group and compared to the baseline values.

## Results

The search identified 20 papers including conference proceedings that reported data from 24 studies. Studies evaluating pharmacotherapy and pheromonatherapy were included. A total of 15 studies were excluded from the analysis for reasons detailed in Table 1. Hart *et al.* [21] evaluated two active treatments in their study, for the purpose of this analysis each treatment group was considered separately. This left a total of 10 studies for analysis (see: Diagram S1).

**Table 1:** Excluded studies and main reason for exclusion from the analysis.

STUDY REFERENCE	EXCLUSION CRITERION
Schwartz S [23]	Case study
Pageat P [24]	Cats showing sexual spraying
Pageat P [25], Pageat P and Tessier Y [26], Pageat P and Tessier Y [27], Seksel K. and Lindeman MJ [17], White JC and Mills DS [28], Kroll T and Houpt KA [29]	Incomplete description of the materials and methods, with raw data or only descriptive statistics. (conference abstracts), preventing further evaluation
Mills DS and White JC [30]	Follow up study
Marder AR [31]	Not peer reviewed
Hart BL [13] Cooper L and Hart BL [15], Hart BL, Eckstein RA, Powell KL and Dodman NH [16]	Included cats showing horizontal spraying

Two types of trial design were noted, randomised control trials (RCT) and one group (uncontrolled) pre-post design. Four RCT's were identified; this group included one study with a positive control [21] and three studies of treatment against a placebo control group [6], [18], [19]. One of the RCT studies did not provide raw data for the placebo control group [19] thus only the data from the treated group could be used in the analysis. Four studies were open-label one-group pre and post designs [2], [5], [32], [33]. One study included a within subjects control group [1].

Study quality varied (Table 2), four out of the ten studies were double blind randomised controlled trials and thus rated as 1b [6], [18], [19], [21]; although two of the studies from this group had results with wide confidence intervals [6], [21], and 1b level of evidence is typically considered to be a randomised controlled study with a narrow confidence interval [20]. The remaining six studies were open label trials, with the exception of Dehasse [1] and therefore were classed as providing level 4 evidence (equivalent to case series and poor quality cohort and case control studies, and so with a higher risk of bias).

**Table 2:** Information from each study including type of intervention, trial design, blinding and agreed level of evidence according to the Oxford Centre for Evidence-based Medicine Levels of Evidence.

STUDY	INTERVENTION	DESIGN	BLINDING (SELF REPORT)	BLINDING (INVESTIGATOR)	LEVEL OF EVIDENCE
Frank *et al*. [2]	F3 pheromone spray	One-group pre and post design	No	No	4
Hunthausen [32]	F3 pheromone spray	One-group pre and post design	No	No	4
Mills and Mills [6]	F3 pheromone diffuser	RCT	Yes	Yes (pers. comm..)	1b -
Ogata and Takeuchi [5]	F3 pheromone spray	One-group pre and post design	No	No	4
Dehasse [1]	Clomipramine	One-group, placebo vs. treatment	Yes	No	4
Hart *et al*. [21]	Clomipramine	RCT active control	Yes	Yes	1b -
Hart *et al*. [21]	Fluoxetine	RCT active control	Yes	Yes	1b
King *et al*. [19]	Clomipramine	RCT	Yes	Yes	1b
Landsberg and Wilson [33]	Clomipramine	One-group pre and post design	No	No	4
Pryor *et al.* [18]	Fluoxetine	RCT	Yes	Yes	1b

**RCT (randomised, controlled trial), “-” denotes a single result with a wide Confidence Interval.**

### Types of participants

Cats of both sexes neutered and entire were included, notably all of the studies evaluating pharmacological interventions only recruited neutered cats with the exception of one study [1]. Breed was not always stated so it can not be assumed that all breeds are represented. The age of the participant was not always stated, when reported cats ranged in age between 4 months and 16.5 years.

#### Home environment

The home environment of the feline participants varied both within and between studies. All cats were studied in their home environment, this was usually the household although, Ogata and Takeuchi [5] reported that two cats from their study population spent a proportion of time in a cage. In all reports the cats were managed either as indoor only cats or indoor cats with some degree of outdoor access.

Cats were recruited from single and multi-cat households, with the exception of Dehasse [1] all authors reported that they recruited from multi-cat households. The number of cats living within each multi-cat household varied, for example, Frank *et al*. [2] reported a range of 2–14 cats per household with a mean of 4.5 and a median of 3. A proportion of studies only recruited cats from multi-cat households with ≤ 4 cats [18], [21], [33].

#### Description of spraying behaviour

All studies reported that cats showed the classic signs described by Dehasse [1] of a cat spraying a vertical surface. Not all authors noted the duration of the problem behaviour, when it was reported the minimum duration prior to entering a study was 5 days and the maximum was 10 years. The studies evaluating pheromonatherapy appeared more likely to report on the duration of the behaviour. With the exception of Hunthausen [32] all authors that stated they recruited from multicat households ensured that the spraying cat was identified.

#### Veterinary screening

Cats entering the study were either veterinary referrals or screened for underlying physiological complications that could influence spraying behaviour or interfere with the evaluation of the treatment. Cats were enrolled with evidence of feline lower urinary tract disease and other concurrent medical disorders (see Frank *et al.* [2] and Landsberg and Wilson [33] for examples).

#### Behavioural advice offered during the study

The tendency to offer behavioural advice differed between studies. Frank *et al* [2]; Landsberg and Wilson [33]; Mills and Mills [6]; Ogata and Takeuchi [5] stated that no specific behavioural advice was offered whereas, Hart *et al.* [21]; King *et al.* [19]; Pryor *et al.* [18] advised owners to employ basic environmental management such as ensuring urine marks were cleaned with an appropriate enzyme based cleaner and keeping litter boxes clean.

#### Duration of intervention period

In the main, the pharmaceutical interventions were administered for a longer duration than the pheromone interventions. The intervention period for pheromone treatment was consistently reported after 28 days in comparison to a mean of 67 days for the pharmacotherapy with a minimum of 7 days [1] and a maximum of 112 days [21].

### Placebo effect

Two randomized controlled studies included a negative control group (placebo) [6], [18]. Pryor *et al.* [18] examined the influence of fluoxetine and reported that 0/8 cats in the placebo group ceased or reduced spraying by at least 90%. Mills and Mills [6] evaluated the use of a synthetic feline facial pheromone F3 diffuser, and noted that in the placebo group 4/12 cats ceased or reduced spraying by at least 90%. (Table 3). Heterogeneity was not significant between the two studies (Cochran Q test P>0.10), so a global effect was estimated.

**Table 3:** Summary data for the estimated placebo effect.

Study name	Number of events	Total Effective	Event rate (%) with 95% CI
Pryor *et al.* [18]	0	8	
Mills and Mills [6]	4	12	
**Global estimate**	**4**	**20**	**0.20 (20%) [0.03**–**0.38]**

**Heterogeneity: P>0.10.**

The global estimated placebo effect was of 0.20 (20% of cats ceased or reduced urine spraying by at least 90%) with a 95% confidence interval of [0.03; 0.38]. The global placebo effect generated was then used in subsequent comparisons.

### Primary outcome: All studies; Cessation or reduction of at least 90%

**Figure 1:**
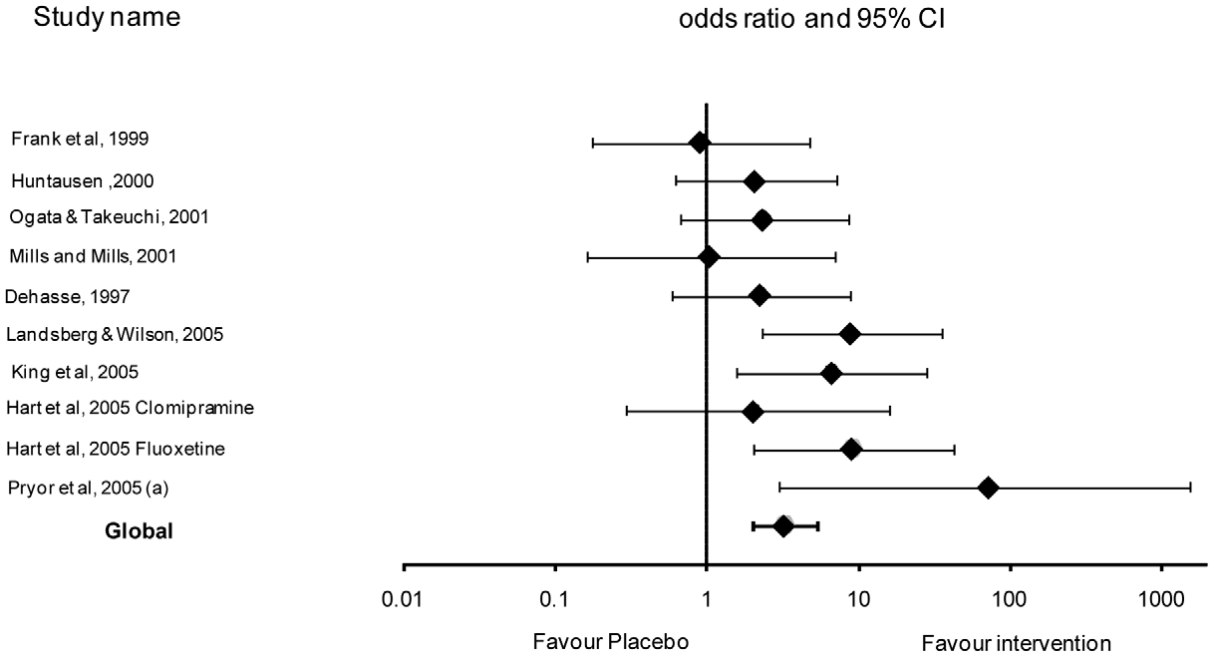
Effect of each study and global effect (OR ± CI 95%) – log scale (x axis).

When all studies were analysed the effect of intervention was significant compared to placebo (p<0.0001). The rate of cessation or reduction in urine spraying of at least 90% was significantly improved by 3.16 in the case of intervention compared to placebo (Odds ratio  = 3.16, 95% confidence limits [1.94–5.14]) (Table 4 and Figure 1). Heterogeneity between studies was not significant (P>0.10).

**Table 4:** Primary outcome All studies; cessation and reduction of at least 90%.

Study name	Intervention	Total N	Event rate	Odds ratio with 95% CI and significance of global estimate
Frank *et al.* [2]	F3 Pheromone spray	17	0.18	0.86 [0.16–4.51]
Hunthausen [32]	F3 Pheromone spray	54	0.33	2.00 [0.58–6.86]
Ogata & Takeuchi [5]	F3 Pheromone spray	36	0.36	2.26 [0.62–8.21]
Mills and Mills [6]	F3 Pheromone diffuser	10	0.20	1.00 [0.15–6.67]
Dehasse [1]	Clomipramine	26	0.35	2.12 [0.54–8.26]
Landsberg & Wilson [33]	Clomipramine	25	0.68	8.50 [2.14–33.81]
King *et al.* [19]	Clomipramine	18	0.61	6.29 [1.48–26.76]
Hart *et al.* [21]	Clomipramine	6	0.33	2.00 [0.27–15.08]
Hart *et al.* [21]	Fluoxetine	16	0.69	8.80 [1.92–40.34]
Pryor *et al.* [18]	Fluoxetine	9	1.00	69.67 [3.37–1440.21]
**Global estimate**				**3.16 [1.94–5.14] P<0.0001**

Heterogeneity: P>0.10.

**Figure 2:**
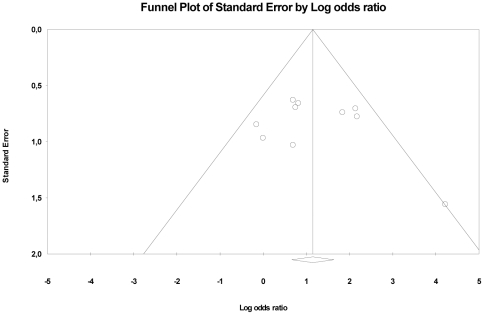
Funnel plot of the association between the estimated effect size and its standard error in individual studies.

There was no evidence of publication bias from visual inspection of the funnel plot (Figure 2).

### Primary outcome: Analysis by intervention type: fluoxetine

**Figure 3:**
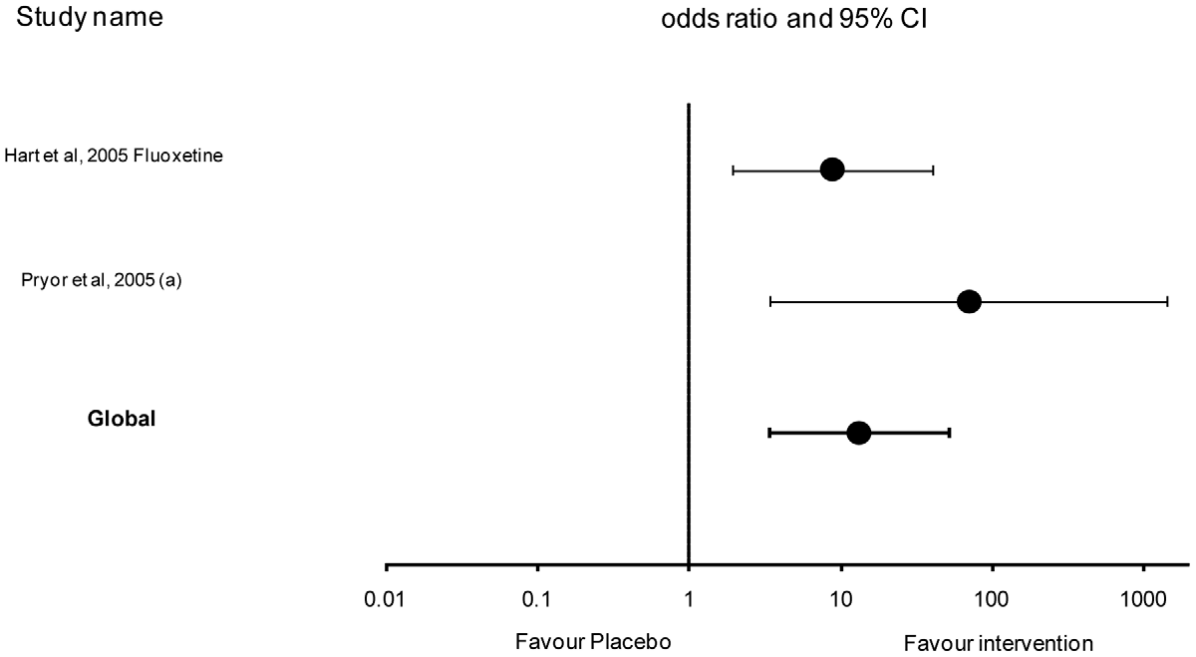
Effect of fluoxetine studies and global effect (OR ± CI 95%) – log scale (x axis).

Two studies were included in the analysis that evaluated fluoxetine. Hart *et al.,* [21] evaluated fluoxetine use over a 16 week period and Pryor *et al.* [18] over 8 weeks. Heterogeneity between studies was not significant (Cochran Q test P>0.10) thus the fixed effects model was used. The number of cats that ceased or reduced urine spraying by at least 90% was significantly (P<0.001) associated with the use of fluoxetine (OR 13.36, 95% CI [3.43–52.06]); (Table 5 and figure 3).

**Table 5:** Primary outcome (Fluoxetine studies); cessation and reduction of at least 90%.

Study name	Intervention	Total N	Event rate	Odds ratio with 95% CI and significance of global estimate
Hart *et al.* [15]	Fluoxetine	16	0.69	8.80 [1.92–40.34]
Pryor *et al.* [18]	Fluoxetine	9	1	69.67 [3.37–1440.21]
**Global estimate**				**13.36 [3.43–52.06] P<0.0001**

Heterogeneity: P>0.10.

### Primary outcome: Analysis by intervention type: clomipramine

**Figure 4:**
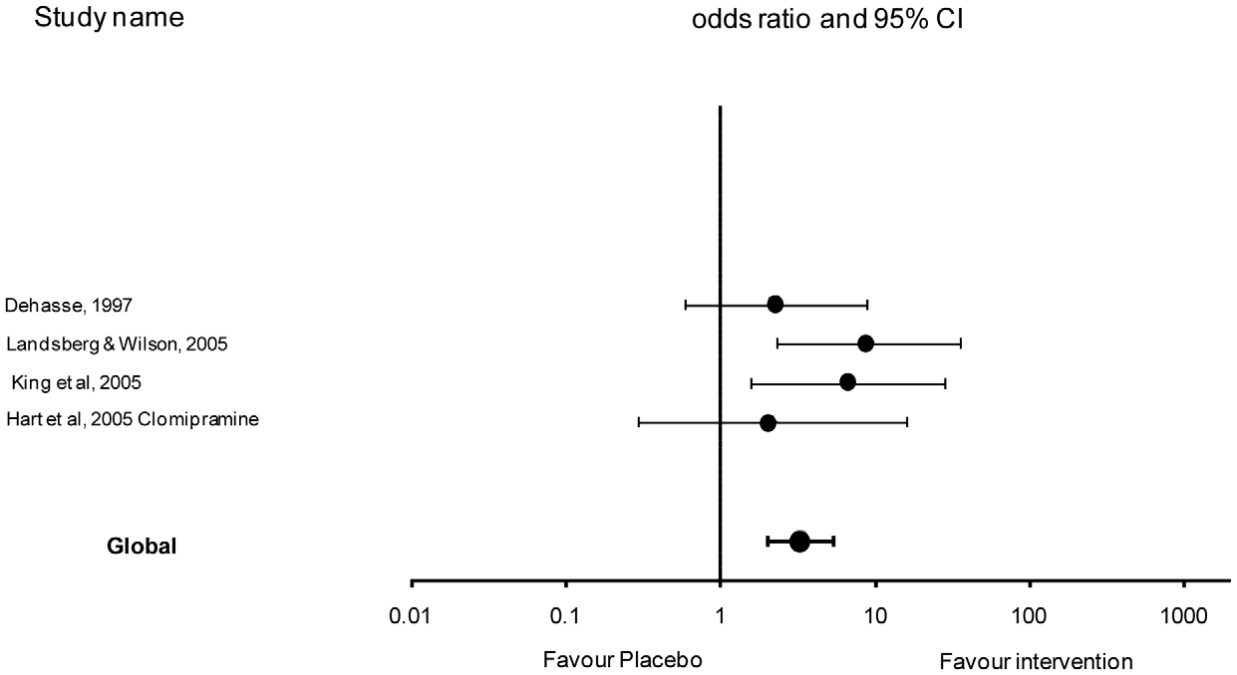
Effect of clomipramine studies and global effect (OR ± CI 95%) – log scale (x axis).

Four studies evaluated clomipramine use [1], [19], [21], [33]. There was no heterogeneity between studies (Cochran Q test P>0.10). There was a significant (P<0.001) association between clomipramine use and the number of cats that cease or reduce urine spraying by at least 90% (OR 4.23, 95% CI [2.00–8.93]): (Table 6 and figure 4).

**Table 6:** Primary outcome (Clomipramine studies); cessation and reduction of at least 90%.

Study name	Intervention	Total N	Event rate	Odds ratio with 95% CI and significance of global estimate
Dehasse [1]	Clomipramine	26	0.35	2.12 [0.54–8.26]
Landsberg & Wilson [33]	Clomipramine	25	0.68	8.50 [2.14–33.81]
King *et al*. [19]	Clomipramine	18	0.61	6.29 [1.48–26.76]
Hart *et al.* [21]	Clomipramine	6	0.33	2.00 [0.27–15.08]
**Global estimate**				**4.23 [2.00–8.93] P<0.0001**

Heterogeneity: P>0.10.

The duration of the treatment period varied between the studies (Dehasse [1] 7 days; Hart *et al.* [21], 16 weeks; Landsberg & Wilson [33], 4 weeks and King *et al.,* [19], 12 weeks).

### Primary outcome: Analysis by intervention type: Synthetic feline facial pheromone F3

**Figure 5:**
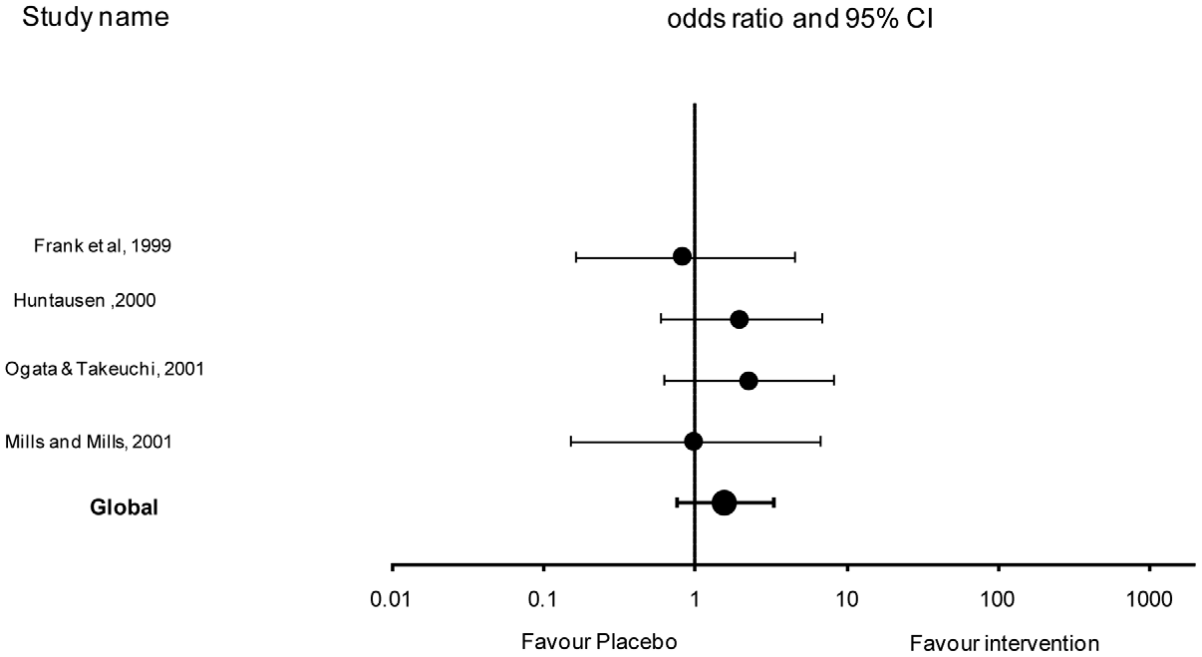
Effect of synthetic facial pheromone F3 studies and global effect (OR ± CI 95%) – log scale (x axis).

Four studies were included in the analysis that evaluated the use of the synthetic feline facial pheromone F3 over a four week treatment period [2], [5], [6], [32]. Heterogeneity was not evidenced between studies (Cochran Q test P>0.10). The analysis showed that the number of cats that ceased or reduced urine spraying by at least 90% was not significant (P = 0.21) but in favour of the synthetic feline facial pheromone F3 (OR = 1.60) with a 95% confidence interval of [0.77–3.30]: (Table 7 and figure 5).

**Table 7:** Primary outcome (Synthetic feline facial pheromone F3 studies); cessation and reduction of at least 90%.

Study name	Intervention	Total N	Event rate	Odds ratio with 95% CI and significance of global estimate
Frank *et al.* [2]	F3 Pheromone spray	17	0.18	0.86 [0.16–4.51]
Hunthausen [32]	F3 Pheromone spray	54	0.33	2.00 [0.58–6.86]
Ogata & Takeuchi [5]	F3 Pheromone spray	36	0.36	2.26 [0.62–8.21]
Mills and Mills [6]	F3 Pheromone diffuser	10	0.20	1.00 [0.15–6.67]
**Global estimate**				**1.60 [0.77–3.30] P = 0.21**

Heterogeneity: P>0.10.

### Secondary outcome: Synthetic feline facial pheromone F3 and Reduction in spraying

Three studies were included in the analysis of the effect of four weeks' of pheromonatherapy on urine spraying (Table 8). No heterogeneity was reported between the studies (Cochran Q test P>0.10) and a fixed effect model was adopted. There was a significant association (P<0.05) between use of synthetic feline facial pheromone F3 and a reduction in urine sprays, there was an overall event rate of 0.92, 95% CI [0.85–0.98] (Table 8).

**Table 8:** Secondary outcome.

Study name	Number of events	Total Effective	Event rate (%) with 95% CI and significance of global estimate
Frank *et al.* [2]	15	17	
Ogata and Takeuchi [5]	33	35	
Mills and Mills [6]	9	10	
**Global Outcome**	57	62	**0.92 (92%) [0.85–0.98] P<0.05**

Heterogeneity: P>0.10.

The influence of synthetic feline facial pheromone F3 (Feliway spray and Feliway diffuser): Number of cats that reduce spraying after treatment.

### Follow up

Six out of ten studies collected data on urine spraying after initial treatment. Duration of the follow up period varied between 3 days [1] and five months [33]. Two studies evaluated pheromone use and reported no statistical evidence of a relapse rate, four weeks after withdrawal of the 4 week-treatment [2], [5]. The remaining four studies all evaluated pharmacotherapy use. Dehasse [1] evaluated the use of clomipramine and reported no evidence of relapse after a period of 3 days after 7 days treatment. After fluoxetine withdrawal for either 4 [18] or 8 weeks [21] there was an increase in spraying rate in comparison to the treatment phase for some cats, with others returning to baseline levels. A more typical follow up period of 5 months was reported by Landsberg and Wilson [33]. They noted that after four week's of clomipramine treatment that only one quarter of the cats did not require medication to control the spraying.

## Discussion

Ten studies were identified as suitable to include in this review based on pre-determined inclusion criteria. However, the quality of evidence available was variable. Half of the studies were double blinded, randomised controlled trials whereas the remaining studies were open label, one-group pre and post designs. Randomised controlled trials with adequate allocation of treatments and masking are considered to provide high quality evidence as bias is minimised. In contrast, open label designs without a control group are generally considered to provide lower confidence in the quality of evidence as they are potentially open to bias due to pre-conceived ideas or misconceptions. To minimise any inherent risk from bias, appropriately masked and randomised, controlled trials are advocated, but they are not always feasible or available, especially in the early phases of development of a product. In general, perceived quality of study design was not associated with intervention type and heterogeneity was generally low, being minimal within the pheromone treatment group (Table 8) even though only one of the studies in this group was a randomised double blinded, controlled trial [6].

Overall, the results suggest that adjunctive interventions beyond general management advice and placebo significantly improved the probability that urine spraying either ceased or reduced by at least 90%. The sustained use of fluoxetine had the largest reported effect. However, only two relatively small studies contributed to this result, with one of these, Pryor *et al.* [18] reporting a complete resolution or at least a 90% reduction in behavioural signs in all nine cats within the treated group. Further evaluation of this treatment is required to establish whether the results can be replicated with larger sample sizes. The evidence presented for clomipramine was more variable with only two out of the four studies [19], [33] clearly different to placebo (Figure 1). The result seems not to relate clearly with daily dosage as Dehasse [1] administered the highest dose of 5mg per cat per day whereas King *et al.* [19] administered the lowest dose at 0.25–0.5mg/kg/d. This may reflect variation in the populations of cats or disparities between study designs such as, an interaction between dosage versus duration of treatment. Within this treatment group, it is worth noting that the largest reported effect size was from the only open label trial [33] this may suggest that knowledge of the treatment influences reliability of the outcome number of spraying marks reported by owners.

The synthetic feline facial pheromone F3 presented as a spray or a diffuser had, on average, little effect on the probability of cessation or at least a 90% reduction in behavioural signs after 4 weeks only. In contrast, a large, effect was detected when considering the number of cats that reduced spraying by week four of treatment in comparison to baseline. This suggests that pheromones do reduce the overall incidence of spraying after only four weeks. In comparison, the pharmacological studies consistently emphasise their effect after 8–16 weeks in the case of fluoxetine and up to 16 weeks in the case of clomipramine, there is therefore a need for longer term studies reporting on pheromonatherapy efficacy on durations equivalent to those used to establish efficacy for pharmaceutical treatments using comparable criteria, such as the “at least 90% reduction threshold” which is reported here. Results from such studies would allow more direct comparison with pharmacological agents. Larger field studies comparing pheromonatherapy with pharmacotherapy over the more typically prolonged period used in pharmacological studies would also be of particular value in highlighting any compliance differences between the two strategies in practice, since the oral dosing required for effective medication is often considered to be a barrier to the use of medication and favour the use of pheromonatherapy. However there are currently no studies to support or refute this suggestion in this incidence.

Recurrence rate is likely to be determined by individual factors within a given case and duration since treatment cessation. In the latter case it is important to distinguish between relapses associated with ongoing treatment or the withdrawal of treatment before complete resolution of the original problem. In addition, animals may become exposed to new stressors which instigate the problem again (technically, these should not be considered relapses as they are a new incidence of the problem). Further evaluation of recurrence rate is required for all interventions and this review highlights some inconsistencies between studies. For example, the duration of time between treatment cessation and subsequent follow up was highly variable (between 3 days –20 weeks). If it is accepted that three days can not be considered normal for monitoring follow up [1] duration of follow up varied between 4 and 20 weeks.

Frank *et al* [2] and Ogata and Takeuchi [5] both reported an initial reduction or cessation in spraying behaviour after pheromone treatment. The latter authors also independently note that four weeks after treatment withdrawal that a return to baseline levels of the problem was not evident. This result concurs with a longer term study [30] that reported on spraying behaviour 10 months after a five week treatment period with a pheromone spray [28]. Forty three owners were contacted, none of the owners had been using the treatment on a daily basis and thirteen owners still used the intervention intermittently. Although 77% of cases were considered to be under adequate control from the owner's perspective only 6 cats (14%) were not spraying at all at this time, with 27 cats (63%) spraying at a lower rate than at the start of the study. This raises important questions about owner compliance and how success should be evaluated, since an owner may make a trade off between a certain acceptable rate of spraying and the effort required to reduce it. Therefore, controlled laboratory studies may be important in determining the objective potential of these interventions, rather than their field efficacy. With every treatment, an increase in spraying frequency was noted for a majority of cats [18], [28], and in one report for all cats [21] after the treatment intervention ceased and this deserves further investigation, although in at least one report [28] the majority of cats were spraying at a lower level compared to baseline 10 months after treatment.

When comparing studies it was evident that certain differences existed between the feline populations sampled and this tended to correlate with the type of intervention under evaluation, three notable discrepancies are discussed.

Five out of the 6 pharmacological studies only sampled neutered cats, whereas non-neutered cats were included in the pheromone studies. How much this impacted on the individual studies results is unclear given that all cats sampled were classed as reactional sprayers rather than sexual sprayers [1]. However, it is often recommended as a first line of treatment to neuter cats that are persistently urine spraying [13].

Overall, the maximum number of cats living in each household was greater for the pheromone studies with a range of between 1–31 cats per household. The risk of inter cat aggression within the household is likely to increase in line with an increasing feline population due to limited space and access to resources [34], [35]. Frank *et al.* [2] and Ogata and Takeuchi [5] both note that pheromone treatment success was reduced within multi-cat households with notable inter-cat aggression. The impact of this on pharmacological interventions remains unknown.

Simple environmental management can reduce the weekly spraying rate [12]. However, only owners taking part in certain trials evaluating the pharmacological interventions were asked to implement management changes whilst administering the intervention under evaluation. Owners were not asked to implement changes whilst using the synthetic feline facial pheromone F3. Thus the effect of pheromones reported here may be a conservative estimate.

The studies evaluating pharmacological interventions selected their subjects on the basis of more stringent inclusion criteria. These criteria will have ensured a more uniform study population and together with the additional environmental interventions made may have inflated the relative effect reported here. It would be of benefit for future studies that seek to evaluate interventions to use comparable stringent criteria to maximise the specificity of effect.

The studies evaluating pheromonatherapy were more likely to report on the history of the spraying behaviour. However, no associations were found between treatment success and either duration of the problem or number of cats in the household or age of cats [28]. It would be interesting to ascertain whether this also applies to pharmacological interventions.

We recognise that there are limitations associated with the conclusions from the summary effect presented here, given that it was necessary to generate an estimated placebo effect size, but we believe this does at least provide a common benchmark for comparison and the basis for more objective evaluation of treatment. To enable a more comprehensive meta-analysis in the future it is recommended that studies should review trial design and methods in line with these recommendations: (1) trial design should be a blinded RCT; (2) full subject data should be presented or an estimate of effect size for both the treated and placebo groups; (3) the outcome measure should be clearly established and include the number of cats that cease or reduce spraying by at least 90% and also the numbers that reduce by a given amount.

In conclusion, there is good evidence that both pharmacological and pheromonal interventions provide added value for the reduction of urine spraying in the cat. It is worth noting that the most extensive treatment programme described, i.e. the one involving a triple line intervention consisting of psychopharmacology (fluoxetine), environmental modification and a cleaning regime for the longest period of time appears to be the most effective treatment documented to date. This emphasises the potential need for a comprehensive treatment programme for maximum effect. Further studies are required to dissect out the relative importance of each element and their possible synergies with other treatments (e.g the use of pheromonatherapy) in addition. In addition further masked, controlled trials with stricter study population inclusion criteria for at least eight weeks are also required especially in relation to the effect of F3 facial fraction pheromone on feline urine spraying in the household.

## Supporting Information

Diagram S1 PRISMA Flowchart.
(DOC)
