# Supplementary material for: A Meta-Analysis of Studies of Treatments for Feline Urine Spraying
Source: PLoS One. 2011 Apr 15;6(4):e18448. doi: 10.1371/journal.pone.0018448 (PMC3078130; doi:10.1371/journal.pone.0018448)
Supplement: Diagram S1 — PRISMA Flowchart. (DOC) [file pone.0018448.s001.doc]

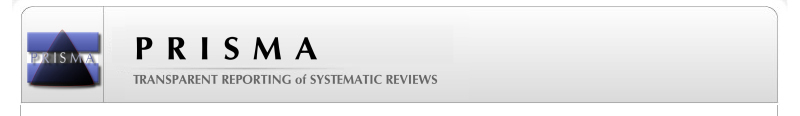
**S1 PRISMA 2009 Flow Diagram**

**Screening**

**Included**

**Eligibility**

**Identification**

Records identified through database searching
(n = 20 )

Additional records identified through other sources
(n = 0 )

Records after duplicates removed
(n = 20 )

Records screened
(n = 9 )

Records excluded
(n =11 )

Full-text articles assessed for eligibility
(n = 9 )

Full-text articles excluded, with reasons
(n = 9 )

Studies included in qualitative synthesis
(n =10 )

Studies included in quantitative synthesis (meta-analysis)
(n = 10 )
